# Case report: Hemangioblastoma in the brainstem of a dog

**DOI:** 10.3389/fvets.2023.1126477

**Published:** 2023-03-22

**Authors:** Kirsten Landsgaard, Samantha St. Jean, Stephanie Lovell, Jonathan Levine, Christine Gremillion, Brian Summers, Raquel R. Rech

**Affiliations:** ^1^Department of Veterinary Pathobiology, School of Veterinary Medicine and Biomedical Sciences, Texas A&M University, College Station, TX, United States; ^2^Department of Small Animal Clinical Sciences, School of Veterinary Medicine and Biomedical Sciences, Texas A&M University, College Station, TX, United States; ^3^Department of Large Animal Clinical Sciences, School of Veterinary Medicine and Biomedical Sciences, Texas A&M University, College Station, TX, United States; ^4^School of Veterinary Medicine, University of Melbourne, Werribee, VIC, Australia

**Keywords:** blood vessel, brain, central nervous system, canine, case report

## Abstract

A 3-year-old castrated male, American Pit Bull Terrier presented to Texas A&M University due to a 3-week mixed cerebellar and general proprioceptive ataxia, circling, head tilt, and dull mentation. Neurologic examination revealed signs of vestibular and mesencephalic dysfunction. Postmortem examination revealed a 1.1 × 1 × 0.8-cm, soft, dark red, well-circumscribed, left-sided mass, extending from the crus cerebri of the midbrain caudally to the pons. Microscopically, the neoplasm was composed of a spindle-shaped interstitial population of cells interspersed between a prominent capillary network, consistent with the reticular pattern of hemangioblastoma. Interstitial cells had strong, diffuse, intracytoplasmic immunolabeling for neuron-specific enolase (NSE) and were variably positive for intracytoplasmic glial fibrillary acidic protein (GFAP). Vascular endothelial cells had strong diffuse, intracytoplasmic immunolabeling for von Willebrand factor (VWF) glycoprotein. To date, only six cases of hemangioblastoma have been reported in canines, five in the spinal cord, and one in the rostral cerebrum. Our case may represent the first canine hemangioblastoma localized to the brainstem.

## Introduction

Hemangioblastomas are rare, low-grade, highly vascular tumors of uncertain histogenesis (1). They have been described in humans, dogs, and sheep. Presenting as expansile central nervous system (CNS) masses, hemangioblastomas produce clinical signs by obliteration and compression of the neuroparenchyma. Most veterinary cases are canine tumors. These include intramedullary (2, 3) and intradural extramedullary in the vertebral canal and, less often, intracranial intra-axial tumors (4). They are rarely found extraneurally in animals, except in sheep (5). Retrospectively, cases within the canine spinal cord are often incorrectly given a diagnosis of capillary hemangioma due to the prominent and conspicuous capillary component, and so may be under-reported (1). Clinical signs can reflect the location and size of the mass. As with most central nervous system (CNS) neoplasms, management is difficult. In spinal cases, surgical removal and chemotherapy have led to improvement in clinical signs in one case (6); however, depending on the depth of invasion, this approach may not be possible. Tumor recurrence has been noted (7).

## Case description

A 3-year-old castrated male, American Pit Bull Terrier dog presented to the Texas A&M Veterinary hospital for the evaluation of a 3-week, progressive course of mixed cerebellar and proprioceptive ataxia, left-sided circling, transient left head tilt, and dull mentation. Neurologic examination revealed delayed postural reactions in the right limbs, delayed oculocephalic reflexes bilaterally, and positional nystagmus, all consistent with central vestibular and mesencephalic dysfunction.

**Figure 1:**
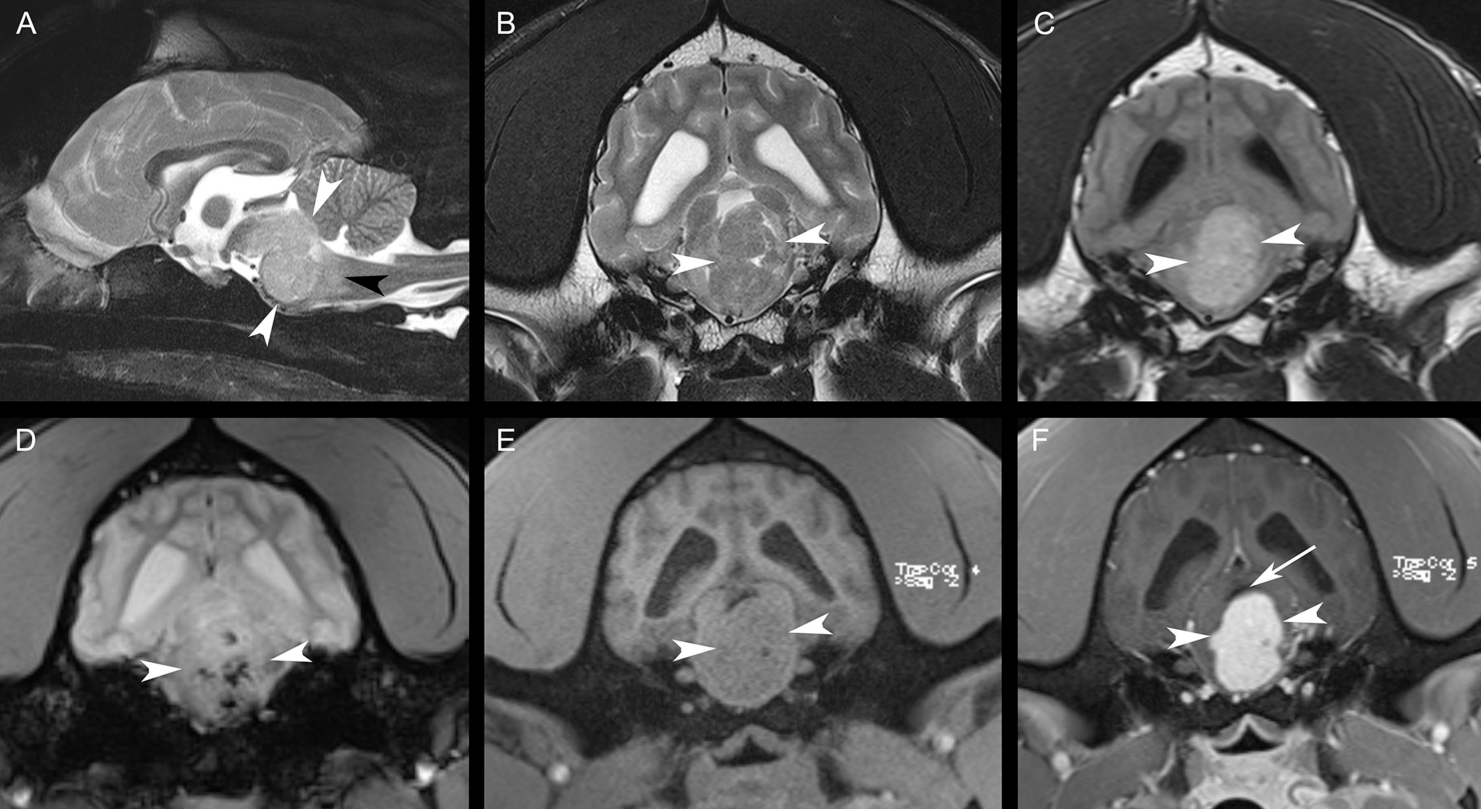
Sagittal T2W fat-suppressed image **(A)**, transverse T2W image **(B)**, transverse FLAIR image **(C)**, transverse T2*W image **(D)**, transverse T1W image **(E)**, and transverse post-contrast T1W image **(F)**. There is a large, strongly contrast-enhancing intra-axial mass [white arrowheads, **(A–F)**] expanding the brainstem with resultant perilesional edema [black arrowhead, **(A)**]. Note the multiple regions of the signal void within the mass in **(D)**. The mesencephalic aqueduct is dorsally displaced and compressed by the mass [white arrow, **(F)**]. The lateral ventricles are mildly asymmetrically dilated in **(B–F)**.

Magnetic resonance imaging (MRI) of the head was performed with the patient under general anesthesia, using a knee coil and 3T magnet (Siemens 3T Magnetom Verio, Malvern, PA). T2-weighted (T2W) transverse, sagittal, and dorsal plane; T2^*^-weighted (T2^*^W) transverse plane; fluid-attenuated inversion recovery (FLAIR) transverse plane; and T1-weighted (T1W) transverse, sagittal, and dorsal plane images of the brain were obtained. Additional T1-weighted (T1W) transverse, sagittal, and dorsal plane images were acquired following intravenous administration of gadobutrol. MRI revealed a strongly contrast-enhancing intra-axial mass expanding the brainstem, which was heterogeneously T2W and FLAIR isointense to hyperintense and T1W isointense to hypointense compared to white matter (Figure 1). Multiple T2^*^W signal voids were identified within the mass, suggestive of hemorrhage (Figure 1). Surrounding the mass, the neuroparenchyma was T2W and FLAIR hyperintense, presumed to represent mild perilesional edema (Figure 1). This mass caused the compression of the cerebellum, narrowing and right dorsal lateral displacement of the mesencephalic aqueduct, and dilation of the ventricular system rostral to the compression (obstructive hydrocephalus) (Figure 1). Prioritized differential diagnoses based on imaging included a high-grade glioma, round cell neoplasia (histiocytic sarcoma, lymphoma), or less likely a granuloma. Due to poor prognosis, the animal was humanely euthanized.

**Figure 2:**
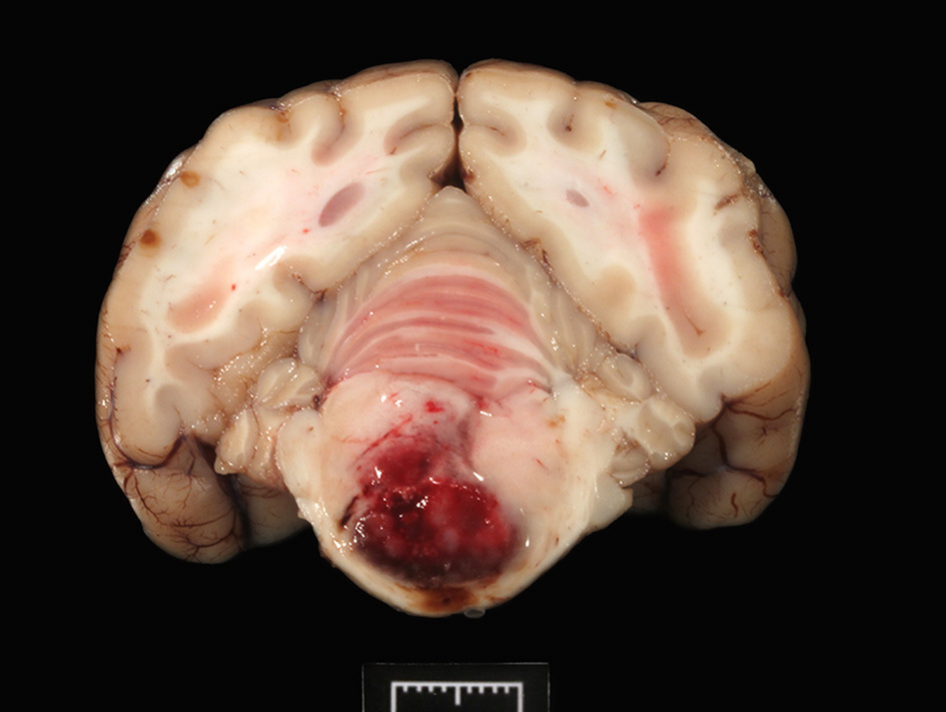
The transverse section of the pons shows a focal, large, bulging hemorrhagic predominantly left-sided mass (1 cm).

**Figure 3:**
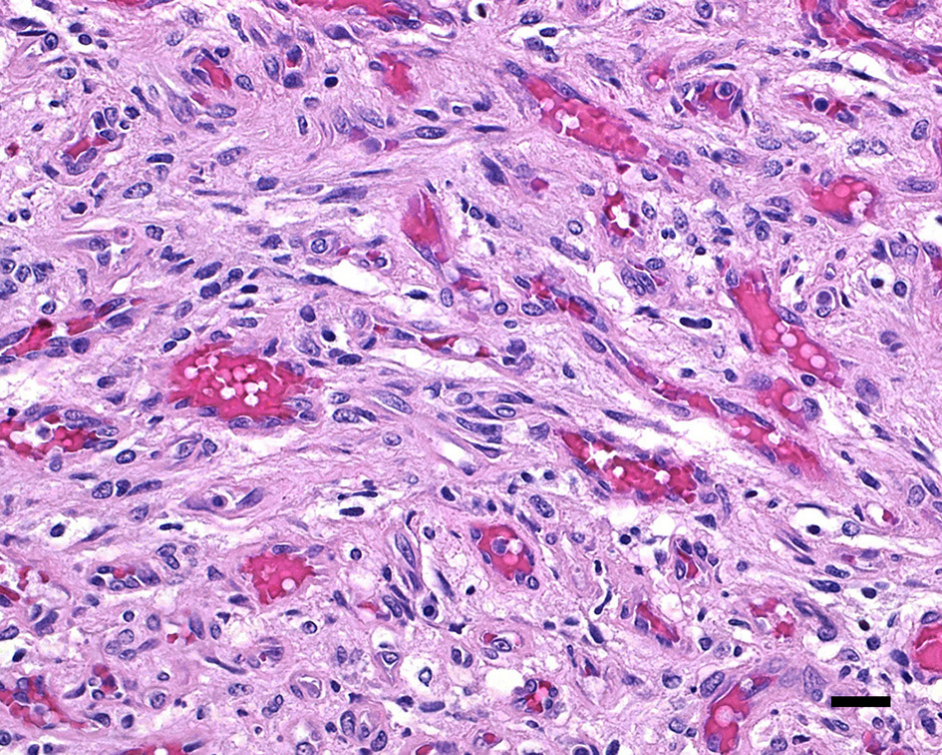
Hemangioblastoma with a prominent network of congested capillary vessels and stellate interstitial cells; 20 μm scale bar, H&E, 40×.

**Figure 4:**
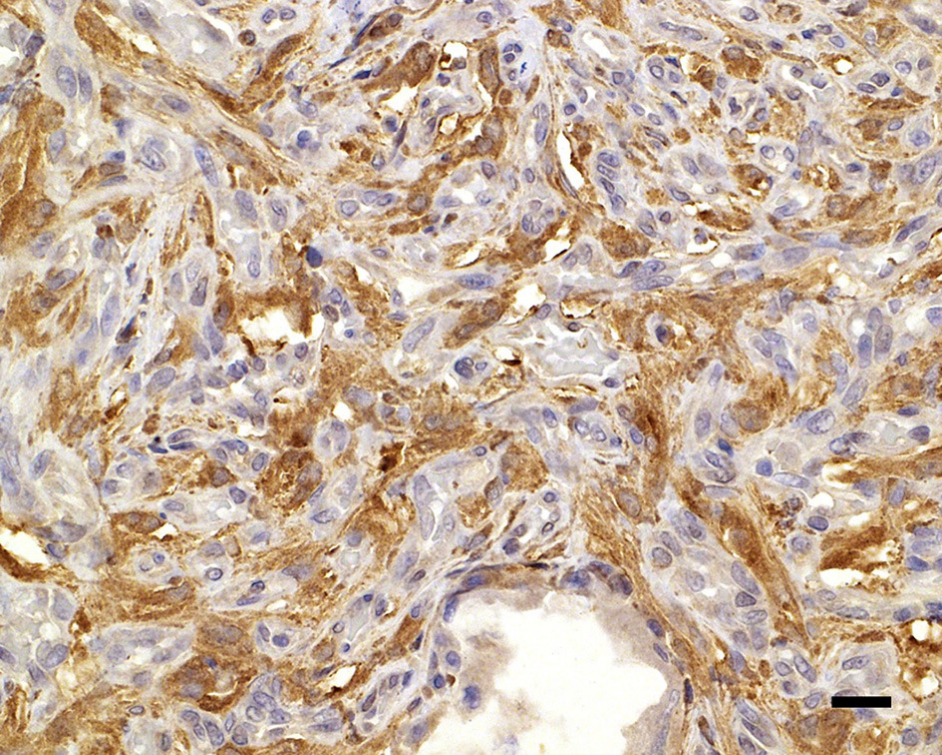
Neuron-specific enolase immunolabels the cytoplasm of the widely dispersed interstitial cells, clarifying cell shapes and sizes; 20 μm scale bar NSE, 40×.

**Figure 5:**
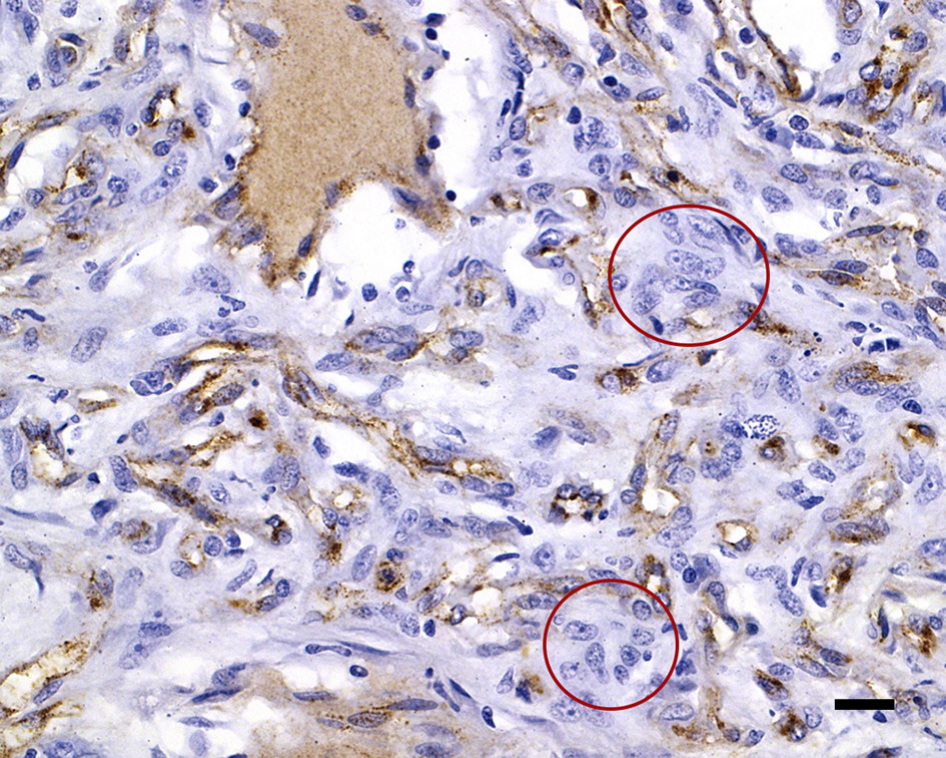
von Willebrand factor immunolabels the capillary endothelial cell cytoplasm of the capillaries, with negative interstitial cells (red circles); 20 μm scale bar vWF, 40×.

**Figure 6:**
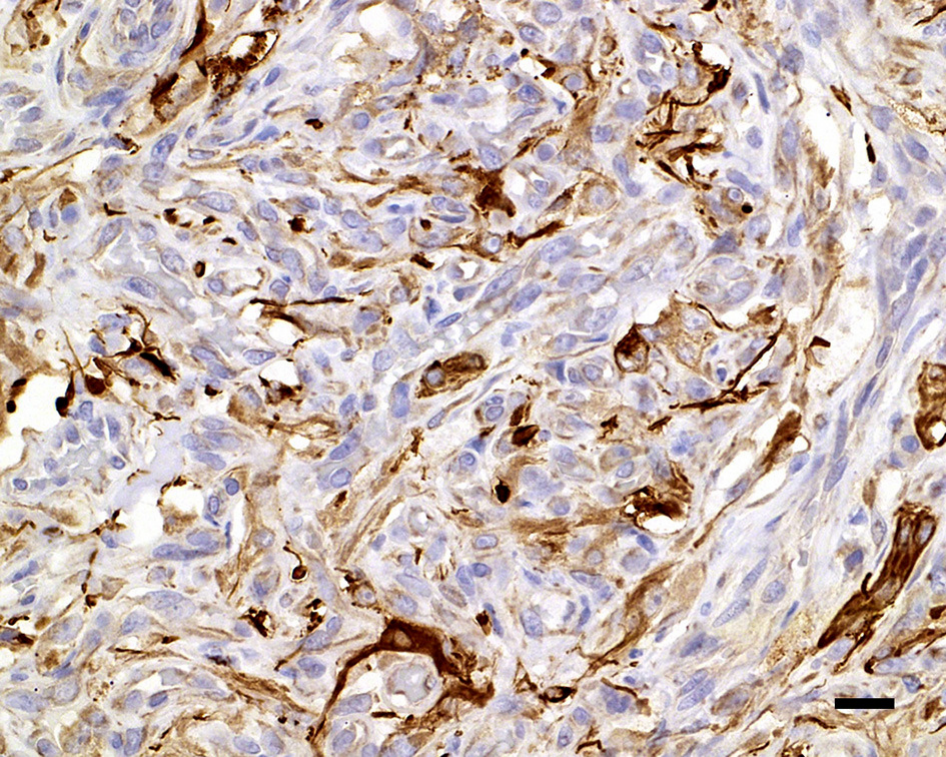
Glial fibrillary acidic protein variably immunolabels the cytoplasm of the interstitial cells; 20 μm scale bar GFAP, 40×.

During necropsy, the ventral aspect of the left side of the brainstem was markedly expanded and soft. An additional finding included a single 24.5-cm, linear, pale tan to white, thin round nematode (*Dirofilaria immitis*) in the pulmonary artery and right ventricle. Following necropsy, the brain was fixed in 10% neutral buffered formalin for 72 h prior to sectioning. A cut section revealed a 1.1 × 1.0 × 0.8-cm gelatinous, soft, well-circumscribed, left-sided mass extended from the crus cerebri of the midbrain caudally to the pons (Figure 2). The sections were routinely processed for histology and stained with hematoxylin and eosin (H&E). Following H&E evaluation, several immunohistochemical markers were requested including neuron-specific enolase (NSE), von Willebrand factor (vWF), and glial fibrillary acidic protein (GFAP) (Table 1). The neoplasm was composed of an interstitial population of cells located between a prominent, congested capillary network (Figure 3). Neoplastic cells had spindle, fusiform to stellate shapes with indistinct cell borders, a moderate amount of eosinophilic cytoplasm, a single oval to elongate nucleus with finely stippled chromatin, and two variably distinct nucleoli. Interstitial cells with clear to mildly vacuolated cytoplasm were scattered throughout the mass. Anisocytosis and anisokaryosis were moderate, and the mitotic count was 7 in 2.37 mm^2^. NSE immunohistochemistry of the interstitial cells had strong, diffuse, intracytoplasmic immunolabeling, and clarified the cell shape and size (Figure 4). Vascular endothelial cells had strong, diffuse, intracytoplasmic immunolabeling for vWF (Figure 5). Interstitial cells also had variably positive intracytoplasmic immunolabeling for GFAP (Figure 6).

**Table 1:** An immunohistochemistry panel performed for the diagnosis of hemangioblastoma in the brainstem of a dog.

**Primary antibody**	**Type**	**Company**	**Dilution**	**Antigen retrieval**	**Method**
Glial fibrillary acidic protein (GFAP)	Rabbit polyclonal	Genetex	1:1,000	Citrate pH 6, pressure cooker	Polymer HRP
					DAB
Neuron specific enolase (NSE)	Mc (mouse anti-human)	Agilent/Dako	1:1,000	Citrate pH 6, pressure cooker	Polymer HRP
					DAB
von Willebrand factor (vWF)	Rabbit polyclonal	Biocare Medical	1:300	Citrate pH6, pressure cooker	Polymer HRP
					DAB

Mc, monoclonal; HRP, horseradish peroxidase.

The tumor effaced part of the reticular formation and the longitudinal fibers of the pons (Figure 2), accounting for the circling (compulsive gait), dull mentation, and proprioceptive ataxia. In humans, hemangioblastomas usually develop in the cerebellum (8), less frequently in the spinal cord, in the fourth ventricle (area postrema), and the brainstem (9, 10), as observed in this case. Unusual locations include the suprasellar region, spinal nerve roots (8), and extraneural tissues such as the kidney, adrenal glands, soft tissues, and bone (9). Grossly, hemangioblastomas appear as red solid and/or cystic masses with variegated yellow to orange areas due to the lipid content in the neoplastic cells (8). Hemangioblastomas are considered benign neoplasms, recurring locally if resection is incomplete; however, malignant hemangioblastomatosis, with distinctive widespread subarachnoid growth, has been reported in people years after complete extirpation of cerebellar hemangioblastoma (11).

Vacuolation of the neoplastic interstitial cells has been noted in some canine cases but may be only focal or not present at all. Hemangioblastoma would imply a poorly differentiated vascular neoplasm and the interstitial cells are believed to be the neoplastic population. It is suggested that neoplastic stromal cells represent pluripotent mesoderm stem cells with features of true “hemangioblasts” that can differentiate into endothelial and hematopoietic cells (9). In this capillary-rich neoplasm, as expected, the background vascular component expresses endothelial markers (vWF, CD31), while the neoplastic stromal cells are positive for a surprising diversity of proteins including NSE, NCAM (neural cell adhesion molecule), GFAP, ezrin (a cytoskeleton-linking structural protein), and inhibin (a gonadal hormone) (12, 13). Other positive immunohistochemical markers in the stromal cells include brachyury and vimentin (8).

In humans, two distinct histologic patterns of hemangioblastoma are recognized: reticular, with a mix of capillaries and stromal cells, as in this dog's case, and cellular, predominantly composed of stromal cells. The neoplasm may be solitary (sporadic) or, in humans, part of the autosomal dominant disorder von Hippel–Lindau disease, which leads to hemangioblastoma of the brain, retina, and other organs (14). Previously, only six cases of hemangioblastoma have been reported in dogs, mostly 6–9 years of age. Five of these cases were within the spinal cord and only one was in the rostral cerebrum. Our case may represent the first canine hemangioblastoma localized to the brainstem and so must be added to the differential diagnosis of intracranial masses in the dog (1).

## Data availability statement

The original contributions presented in the study are included in the article/supplementary material, further inquiries can be directed to the corresponding author.

## Ethics statement

Ethical review and approval were not required for the animal study because the case report is a description of a clinical case. Written informed consent was obtained from the patients/participants for the publication of this case report.

## Author contributions

All authors listed have made a substantial, direct, and intellectual contribution to the work and approved it for publication.
